# Effects of insulin on human pancreatic cancer progression modeled *in vitro*

**DOI:** 10.1186/1471-2407-14-814

**Published:** 2014-11-06

**Authors:** Michelle T Chan, Gareth E Lim, Søs Skovsø, Yu Hsuan Carol Yang, Tobias Albrecht, Emilyn U Alejandro, Corinne A Hoesli, James M Piret, Garth L Warnock, James D Johnson

**Affiliations:** Department of Cellular and Physiological Sciences, University of British Columbia, Vancouver, BC Canada; Department of Surgery, University of British Columbia, Vancouver, BC Canada; Department of Chemical and Biological Engineering, University of British Columbia, Vancouver, BC Canada; Département de génie chimique | Department of Chemical Engineering, Université McGill University, 3610 University Street, Wong Building, Room 4230, Montréal, H3A 0C5 Canada

**Keywords:** Hyperinsulinemia, Pancreatic cancer, PANC1, HPDE, Diabetes, PDAC, Pancreatic ductal adenocarcinoma, AKT, ERK

## Abstract

**Background:**

Pancreatic adenocarcinoma is one of the most lethal cancers, yet it remains understudied and poorly understood. Hyperinsulinemia has been reported to be a risk factor of pancreatic cancer, and the rapid rise of hyperinsulinemia associated with obesity and type 2 diabetes foreshadows a rise in cancer incidence. However, the actions of insulin at the various stages of pancreatic cancer progression remain poorly defined.

**Methods:**

Here, we examined the effects of a range of insulin doses on signalling, proliferation and survival in three human cell models meant to represent three stages in pancreatic cancer progression: primary pancreatic duct cells, the HPDE immortalized pancreatic ductal cell line, and the PANC1 metastatic pancreatic cancer cell line. Cells were treated with a range of insulin doses, and their proliferation/viability were tracked via live cell imaging and XTT assays. Signal transduction was assessed through the AKT and ERK signalling pathways via immunoblotting. Inhibitors of AKT and ERK signalling were used to determine the relative contribution of these pathways to the survival of each cell model.

**Results:**

While all three cell types responded to insulin, as indicated by phosphorylation of AKT and ERK, we found that there were stark differences in insulin-dependent proliferation, cell viability and cell survival among the cell types. High concentrations of insulin increased PANC1 and HPDE cell number, but did not alter primary duct cell proliferation *in vitro*. Cell survival was enhanced by insulin in both primary duct cells and HPDE cells. Moreover, we found that primary cells were more dependent on AKT signalling, while HPDE cells and PANC1 cells were more dependent on RAF/ERK signalling.

**Conclusions:**

Our data suggest that excessive insulin signalling may contribute to proliferation and survival in human immortalized pancreatic ductal cells and metastatic pancreatic cancer cells, but not in normal adult human pancreatic ductal cells. These data suggest that signalling pathways involved in cell survival may be rewired during pancreatic cancer progression.

## Background

The incidence of pancreatic cancer is increasing, in parallel with the obesity and type 2 diabetes epidemics. Despite intense research efforts, the average 5-year survival rate for pancreatic cancer remains below 5%, which underscores the need to identify key risk factors and to develop preventative measures [1–3]. Multiple epidemiological studies have drawn a positive link between high levels of insulin and an increased risk of pancreatic cancer [1, 4, 5]. Obesity and early stage type 2 diabetes are both associated with elevated insulin levels, known as basal hyperinsulinemia [6]. Given that insulin is a powerful mitogen and that its levels likely vary physiologically within the pancreas [7], it is possible that sustained increases in local insulin levels within the pancreas provide increased growth advantages and pro-survival effects in cells within the pancreas [8]. It is therefore imperative to investigate the effects of insulin on different stages of pancreatic cancer progression.

The molecular mechanisms by which hyperinsulinemia may affect pancreatic cancer progression remain incompletely understood, but several studies have demonstrated the importance of the RAS-MEK-ERK pathway and the PI3K-AKT pathway. Over 90% of human pancreatic adenocarcinoma cases involve the KRAS^G12D^ gain-of-function mutation, and this mutation is sufficient to lead to pre-cancerous lesions and rare tumours in mouse models [9]. The KRas^G12D^ mutation leads to constitutive activation of RAF-MEK-ERK and PI3K-AKT cascades to drive uncontrolled growth, proliferation and survival of cancer cells [10]. KRas-driven transformations can be inhibited by expression of dominant-negative Raf-1, MEK or ERK, which all lie downstream of Ras [11, 12]. It has been established that Raf-1 can promote the initiation, transformation and maintenance of neoplastic lesions in some cancer models [13, 14]. Constitutively active AKT can also transform normal mouse pancreatic duct cells into malignant pancreatic cancer cells *in vivo* [15], but the inability of PI3K-AKT inhibition to affect several Ras-driven cancers suggests that KRas acts on multiple pathways in oncogenesis [10, 16, 17].

In the present study, we examined the effects and mechanisms of insulin in three *in vitro* cell models designed to mimic the progression of pancreatic cancer *in vivo*. These cell models were: pancreatic ductal cell cultures, an immortalized human ductal epithelium cell line (HPDE), and an advanced metatstatic human pancreatic ductal cancer cell line (PANC1). We found that high levels of insulin accelerated the proliferation of immortalized and metatstatic pancreatic ductal cells but not primary ductal cells. Furthermore, the molecular signalling mechanisms activated by insulin were distinct in each model, suggesting that these processes may be rewired during the progression of pancreatic cancer. These studies reveal potential mechanisms of insulin-mediated growth and survival effects and provide a better understanding in the etiology of hyperinsulinemia-associated pancreatic cancer.

## Methods

### Human mixed pancreatic exocrine and ductal cell culture

Primary pancreatic exocrine cells that would normally be discarded were obtained from the Vancouver General Hospital (Vancouver, BC) as part of the Human Islet Transplant Program, from cadaver organ donors who had previously provided informed consent. Dr. Warnock’s organ retrieval protocols are approved by the University of British Columbia Clinical Research Ethics Board. Tissues were from 7 donors, males and females between the ages of 32 and 58. Procedures involved in the culturing, dissociating and sorting of primary mixed exocrine and ductal tissue were adapted from published protocols, with minor alterations [18, 19]. Briefly, human ductal cell culture was performed as follows. First, unsorted primary cells, after being dispersed by shaking incubation for 1 hour and trituration with trypsin, were plated (10 × 10^6^ cells) in T-150 flasks, to allow preferential adhesion and removal of fibroblasts. Then, fibroblast-depleted cell suspensions were then seeded in 6-well plates at cell density of 1.5 × 10^6^ cells per well for further treatments. For immunoblot analysis, dissociated mixed-pancreatic exocrine-ductal cells were used. For cell proliferation and cell survival assays, sorted ductal cells were used (CD90 negative population). Prior to insulin treatments, cells were cultured in basal media (CMRL1066, 0.5 mg/L transferrin, 10 mM nicotinamide, 5 μg/L sodium selenium, 0.5% BSA, 2 mM glutamine) for 6 hours, then treated with 0.2, 2, 20, 200 nM of human recombinant insulin (Sigma Aldrich, Missouri, USA), 5 μM GW5074 (Life Technologies, California, USA), or 100 nM Akti-1/2 (EMD Biosciences, Darmstadt, Germany).

### HPDE and PANC1 cell culture and treatment

HPDE cells were kindly provided by Dr. Ming Tsao. HPDE cells between passages 7 to 15 were used, and were cultured in KSF medium as previously described [20], but switched to DMEM for the experiments because KSF medium contains 779.1 ± 87.43 nM insulin as measured by radioimmunoassay. PANC1 cells (ATCC, Manassas, USA) were cultured in DMEM as previously described [21]. For treatments, cells were washed with PBS and starved in 1 mg/ml glucose DMEM for for 6 hours (HPDE cells), or 24 hours (PANC1 cells). Thereafter, the cells were treated with insulin, IGF-1, DMSO, 10 μM GW5074, 10 μM U0126 (Cell Signaling, USA), 200 nM Akti-1/2 or 1 μM wortmannin (EMD Biosciences). These concentrations were chosen based on the literature and were shown to block signalling in PANC1 cells.

### Cell counting and cell survival assays

The number of cells, live-stained with a concentration of Hoechst-33342 (50 ng/ml) that does not affect viability [22], was measured over time using ImageXpress^MICRO^ high content imaging systems (Molecular Devices, Sunnyvale, California, USA). Images were analyzed with Acuity Xpress 2.0 (Molecular Devices). Cell death was measured by quantifying the percentage of cells incorporating propidium iodide (Sigma-Aldrich, 0.5 μg/ml) [23–25]. Cell viability, as indicated by metabolic capacity, was also quantified using the XTT kit (ATCC). Bromodeoxyuridine (BrdU) incorporation (Roche, Basel, Switzerland) was also used to determine proliferation in primary cells as previously described [19, 26].

### Immunoblotting and protein analysis

Cells were lysed and subjected to immunoblotting as previously described [27]. Polyclonal mouse and rabbit secondary antibodies, monoclonal antibodies for insulin receptor, ERK1/2, p-ERK1/2(T202/Y204), AKT, p-AKT(S473), and cleaved caspase 3 were obtained from Cell Signaling. Mouse monoclonal beta-actin antibody was obtained from Novus Biologicals (Littleton, Colorado, USA). Chemiluminescence of the blots was imaged on films that were subsequently scanned. The density of individual bands was quantified using the histogram function of using Adobe Photoshop CS5 after inversion and auto-contrast functions were applied to the whole image. Protein levels were expressed as the fold change relative to control.

### Statistical analysis

All data were analyzed by paired sample t-test, or one-way or two-way ANOVA, followed by post-hoc tests (Dunnett’s or Bonferroni analysis) with Prism (GraphPad, La Jolla, California, USA). Results are presented as mean ± SEM, and are considered significant if the *p-*value was less than 0.05.

## Results

### Baseline abundance of insulin signalling proteins in human primary pancreatic ductal cells, human HPDE cells and human PANC1 cells

**Figure 1:**
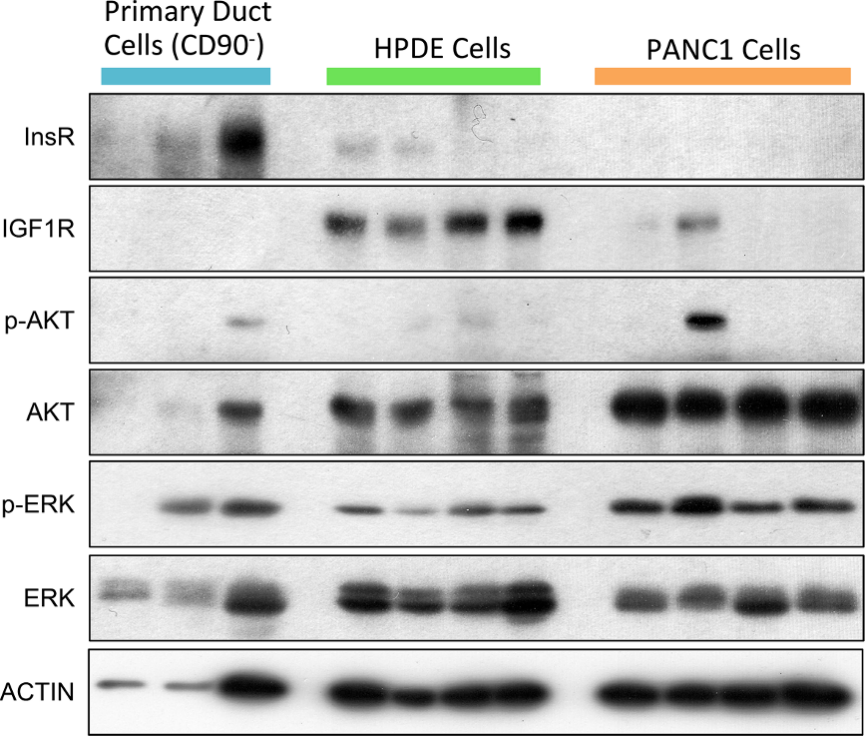
**Baseline abundance of insulin signalling proteins in human primary pancreatic ductal cells, human HPDE cells and human PANC1 cells.** Relative expression of Insulin receptor β (InsRβ), IGF1 receptor (IGF1R), phosphorylated AKT at S473 (p-AKT), total AKT (AKT), phosphorylated ERK (p-ERK), and total ERK (ERK) were examined under basal conditions. From the left to right, there are three biological independent primary ductal cells samples, four HPDE cells samples and four PANC1 cells samples from different passages. Note the uneven loading of primary ductal samples as indicated by the actin loading control, which prevents quantitative comparisons. Other than lanes 1 and 2, every effort was made to load an equal amount of protein into each lane.

Pancreatic ductal adenocarcinoma originates in the exocrine pancreas and progresses to a highly invasive state. In the present study, we attempted to model three states in this progression: normal pancreatic exocrine ductal cells to represent the baseline, HPDE cells to represent a proliferative but non-invasive stage [20, 28, 29], and PANC1 cells to represent a metastatic stage [30, 31]. As a first step in comparing these cell models, we sought to analyze the protein levels of insulin receptor β, IGF1R, AKT and ERK in a small initial pilot western blot study. Notably, protein abundance of insulin receptors appeared to be clearly higher in primary ductal cells than in HPDE or PANC1 cells, even when a fraction of the lysate was loaded (Figure 1). On the other hand, the IGF1R was most highly abundant in HPDE cells (Figure 1). The baseline abundance of downstream signaling proteins, AKT and ERK, was more similar between the models. The total amount of AKT protein appeared to be slightly higher in PANC1 cells. Most cell batches exhibited negligible baseline phosphorylation of AKT on serine 473 (Figure 1). The total amount of ERK tended to be slightly higher in the HPDE cell line, whereas the baseline phosphorylation status of ERK on T402/Y204 was consistently higher in PANC1 cells (Figure 1). While none of these results should be considered quantitative, due to the small nature of the pilot study and the use of antibodies, they do provide some context for the subsequent comparisons of AKT and ERK signaling in response to insulin and IGF1 ligands.

### Insulin signaling in primary human exocrine and ductal pancreas cells

**Figure 2:**
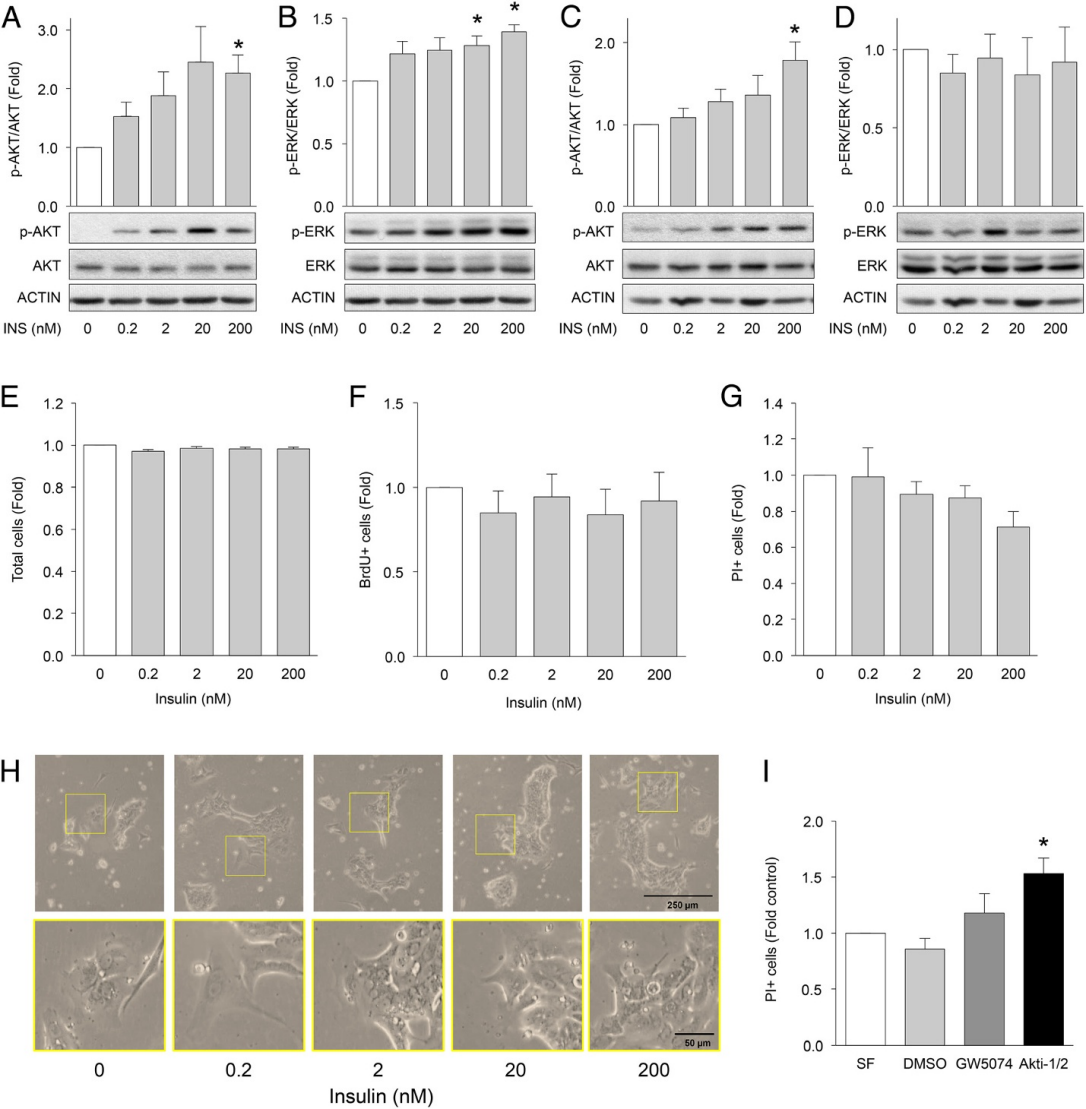
**Effects of insulin on AKT and ERK phosphorylation and cell viability in primary human pancreatic duct cells.** Phosphorylated AKT and ERK were measured in primary pancreatic exocrine cultures treated with the indicated concentrations of insulin for 5 minutes **(A, B)** and 24 hours **(C, D)** (n =3-4) Fold refers to the fold change of sample relative to control at the same time point. **(E)** Quantification of automated cell-counting studies employing live-cell imaging of Hoechst-labeled cell cultures over 60 hours. (n =3). **(F)** Quantification of proliferation by BrdU staining of treated relative to untreated over 3 days (n =4). **(G)** Quantification of the average number of dying/dead treated cells, propidium iodide (PI) labeled, over 60 hours relative to non-treated cells. (n =3). **(H)** Human exocrine cells were exposed to 0, 0.2, 2, 20, 200 nM insulin for 3 days. Bright-field images are representative of 3 cultures. **(I)** Effects of inhibition of RAF1/ERK signalling on PI incorporation with 10 μM GW5074 or AKT signalling with 100 nM Akti1/2 on human primary pancreatic exocrine cell viability (n =3). SF denotes serum free. Repeated Measures ANOVA analyses with Bonferroni’s post-test were performed. *Represents statistical significance of *p* < 0.05 when compared to DMSO control.

To set a baseline for our *in vitro* model of pancreatic cancer progression, we next sought to establish the effects of insulin on normal human pancreatic exocrine-ductal cells. Primary pancreatic exocrine-ductal cells were exposed to a range of insulin doses for 5 minutes (acute) and 24 hours (chronic) and examined for the activation of AKT and ERK signalling. Rapid rises in the phosphorylation of ERK-T402/Y204 and AKT-S473 were detected after acute insulin treatment, most notably with 20 nM and 200 nM insulin treatment (Figure 2A,B). Chronic insulin treatments led to an increase in AKT phosphorylation but not ERK (Figure 2C,D). Proliferative effects of insulin were not observed in sorted primary pancreatic ductal cells (Figure 2E,F). Higher levels of insulin elicited protective effects in sorted primary cells (Figure 2G). Phase contrast microscopy revealed that high doses of insulin altered the granularity, shape, and distribution in of human primary ductal cells in culture (Figure 2H).

The importance of two of the major insulin signalling kinases, ERK and AKT, was evaluated by treating unstimulated cultures with small molecule inhibitors targeting AKT (Akti-1/2) or RAF1 (GW5074), an upstream kinase of ERK. Inhibition of AKT caused a significant increase in PI-positive cells, whereas blocking ERK signalling did not promote cell death (Figure 2I). These data suggest that AKT signalling is critical for the survival of human pancreatic ductal cells, while RAF1/ERK signalling is dispensable, under these basal conditions.

### Insulin signalling in HPDE cells

**Figure 3:**
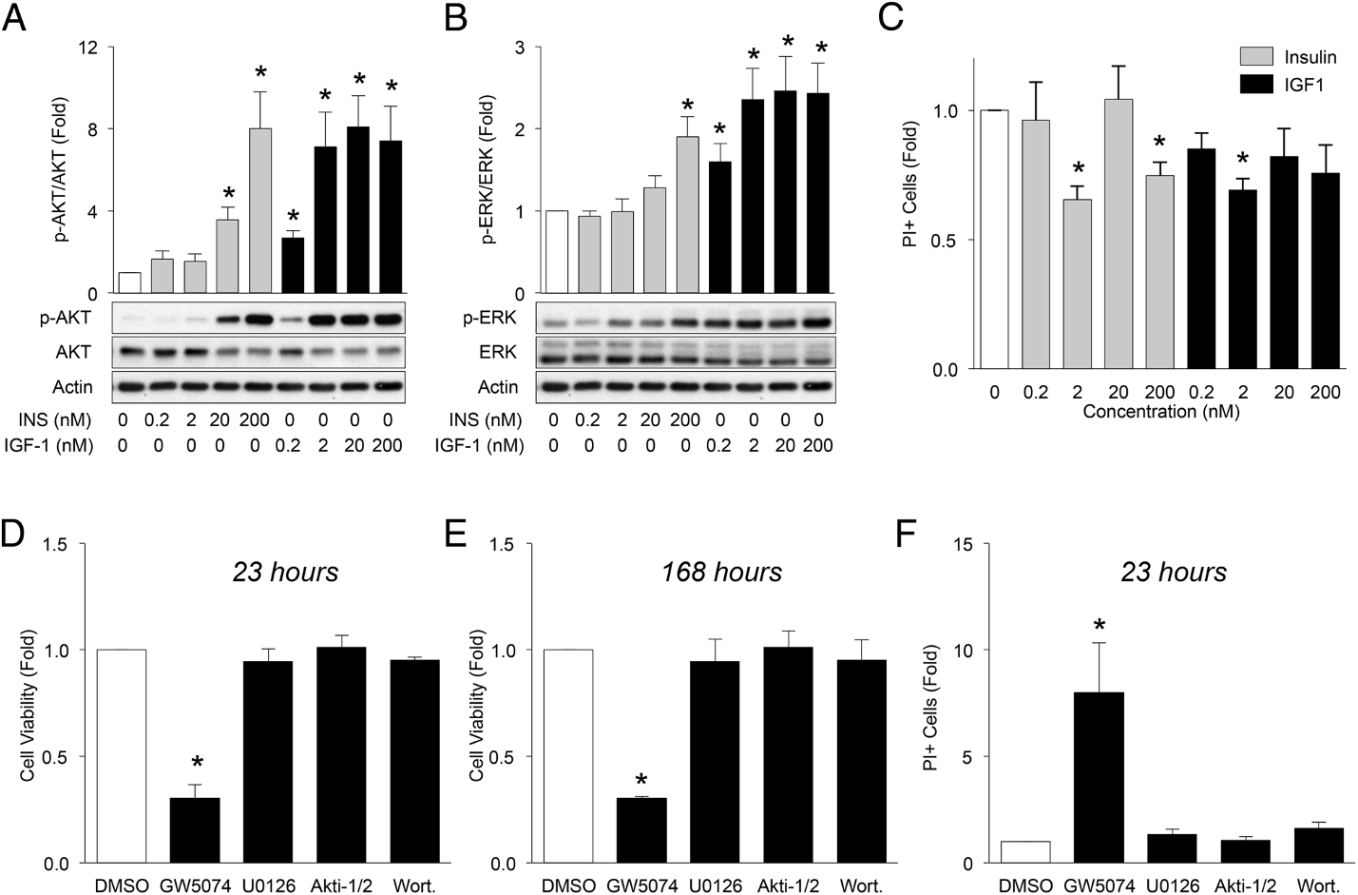
**Effects of insulin on AKT and ERK phosphorylation and cell viability in HPDE cells. (A, B)** Phosphorylated AKT and ERK were measured in HPDE cells treated with a range of insulin and IGF-1 concentrations for 5 minutes (n =10, 8). **(D-E)** Proliferation of HPDE cells was assessed by XTT assay. Briefly, cells were treated and the activated XTT reagent was added at designated time, and the absorbance of Δ(A_475nm_ and A_660nm_) was measured 6 hours post-addition. Insulin or IGF1 was not added in these studies (n =3). **(C, F)** Quantification of cell death was assessed by propidium iodide (PI) incorporation in Hoechst-positive cells. Fold refers to the number of PI positive cells in treatment group relative to control after 23 hours of treatment (n =3). **(A-C)**. Two-tailed paired sample t-tests were performed. **(D-E)** One-way ANOVA analyses with Bonferroni post-test were performed.

HPDE cells are human pancreatic ductal cells that were immortalized by transfection of E6E7 protein from human papilloma virus 16 [20, 28, 29]. Unlike other pancreatic carcinoma cell lines, which commonly reveal homozygous *p16* gene deletion, HPDE cells express normal p16 genotype [29]. As compared to other pancreatic carcinoma cell lines, HPDE cells express relatively lower levels of EGFR, erbB2, TGF-α, HGFR, VEGF and KGF [29]. However, the response profiles of this cell line to insulin and IGF1 have not been reported. This human ductal epithelial cell line has been proposed as an important tool to study pre-cancer or early stages of pancreatic cancer [20]. Here, we used them as a model of proliferating, but not yet cancerous, pancreatic cells. Similar to primary pancreatic ductal cells, HPDE cells displayed responsiveness to insulin, as seen by AKT and ERK phosphorylation (Figure 3A,B). In the absence of serum, insulin as low as 2 nM exhibited protective effects on cell survival in HPDE cells (Figure 3C). Similar results were observed with IGF1, which activates receptors with 75% structural homology. Activation of both insulin and IGF1 receptors has been implicated in pancreatic cancer progression and chemotherapy resistance [32, 33]. Interestingly, HPDE cells were more sensitive to IGF1 than to insulin (Figure 3A,B), but differences in cell survival effects were not observed between these two ligands (Figure 3C). In the absence of serum or exogenous insulin or IGF1, inhibition of RAF1 with GW5074 dramatically decreased HPDE cell viability after only 23 hours (Figure 3D,E). Contrary to what was observed in primary human sorted cells, inhibition of the PI3K-AKT pathway had no effect on HPDE cell viability (Figure 3D-F). Thus, the RAF1 pathway, and not the PI3K/AKT pathway, is required for the maintenance of HPDE cell survival under these basal conditions.

### Insulin signalling in PANC1 cells

**Figure 4:**
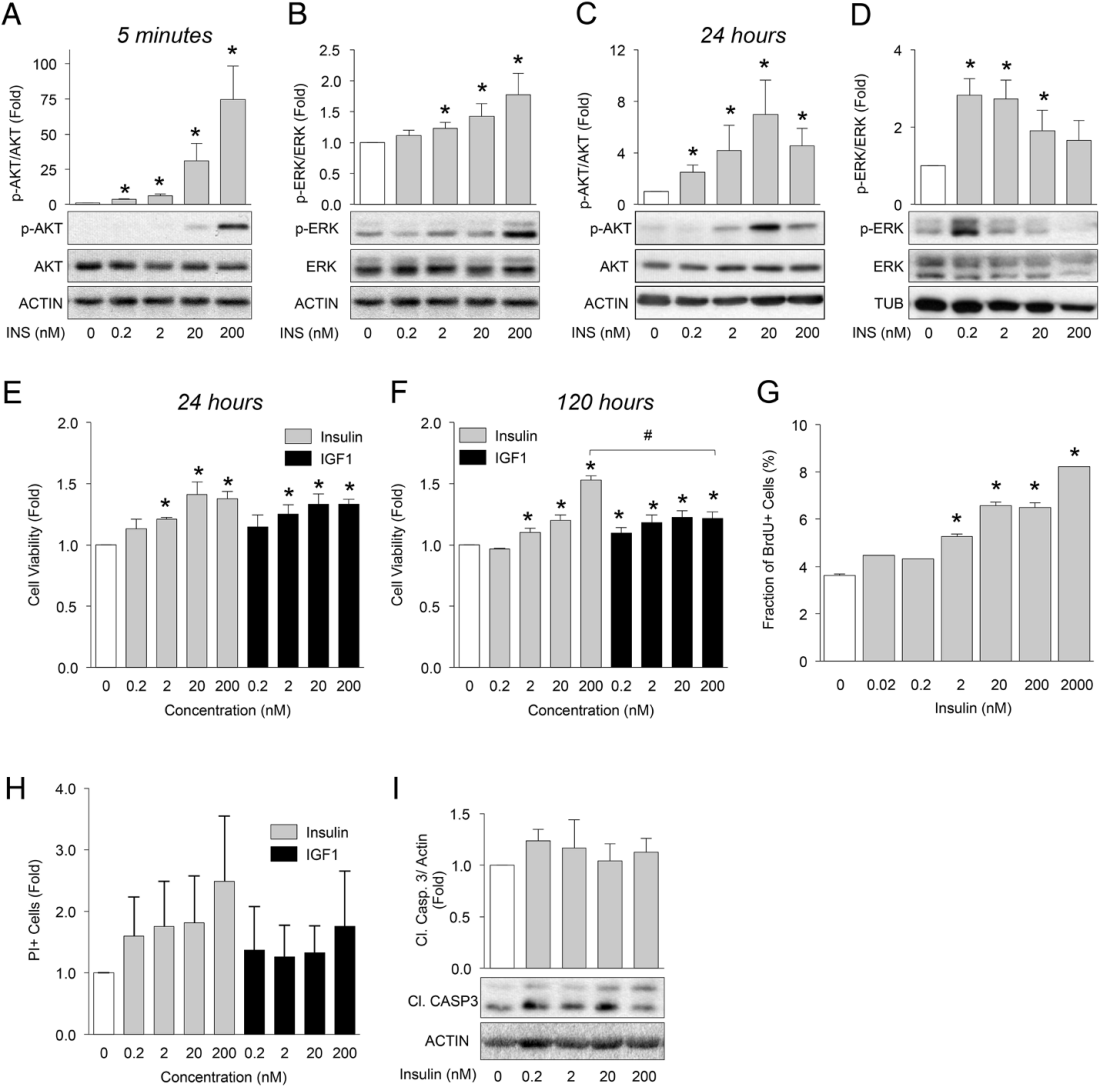
**Effects of insulin on AKT and ERK phosphorylation and cell viability in PANC1 cells.** Phosphorylated AKT and ERK were measured in PANC1 cell cultures treated with the indicated concentrations of insulin for 5 minutes **(A, B)** and 24 hours **(C, D)** (n =7-12). **(E, F)** PANC1 cellular viability was also assessed by XTT at 24 hours or 5 days of incubation, and expressed as fold change in mean absorbance treatment relative to control (n =5-6). Insulin was not added in these studies **(G)** PANC1 cell proliferation measured after 24 hours using BrdU (n =6) Insulin was not added. **(H)** PANC1 cell death measured by propidium iodide incorporation after 48 hours (n =5). Insulin was not added. **(I)** Cleaved caspase 3 was measured after 24 hours (n =5). **(A-F, H-I)** Two-tail paired sample t-test were performed. *Represents statistical significance of *p* < 0.05 when compared to control (0 nM Insulin). # in Figure 4F denotes statistical significance between 200 nM IGF1 and 200 nM insulin when two-tailed paired sample t-test was performed.

The PANC1 cell line was originally isolated from a pancreatic adenocarcinoma containing the constitutively active KRAS^G12D^ mutation, a homozygous p16 deletion and an inactivating p53^R273H^ mutation [30, 31]. This cell line is routinely used to study the late stages of pancreatic cancer. Acute and chronic treatment of PANC1 cells with insulin revealed striking differences in the kinetics and dose–response profiles of AKT and ERK phosphorylation. Several concentrations of insulin tested elicited acute AKT and ERK phosphorylation in these experiments (Figure 4A,B). On the other hand, insulin treatment for 24 hours resulted in maximal AKT activation at the 20 nM dose, without further stimulation by 200 nM insulin. Notably, 24 hours of insulin treatment was only capable of activating ERK at lower doses (Figure 4C,D). We have previously found that lower doses of insulin can be more effective at activating RAF1/ERK and related pathways in pancreatic endocrine cells [25, 26, 34–38] and our recent mathematical model suggests that such low concentrations are present in the human pancreas [7]. Proliferative and protective effects were only observed at higher insulin doses (Figure 4E,F). In PANC1 cells treated for 120 hours, insulin was more effective at promoting cell viability than IGF1. The increase in proliferation induced by insulin was confirmed with BrdU incorporation (Figure 4C). No differences were observed between insulin and IGF1 on cell survival (Figure 4H-I). To the best of our knowledge, this is the first direct comparison of the effects of insulin and IGF1 in pancreatic cancer cells.

**Figure 5:**
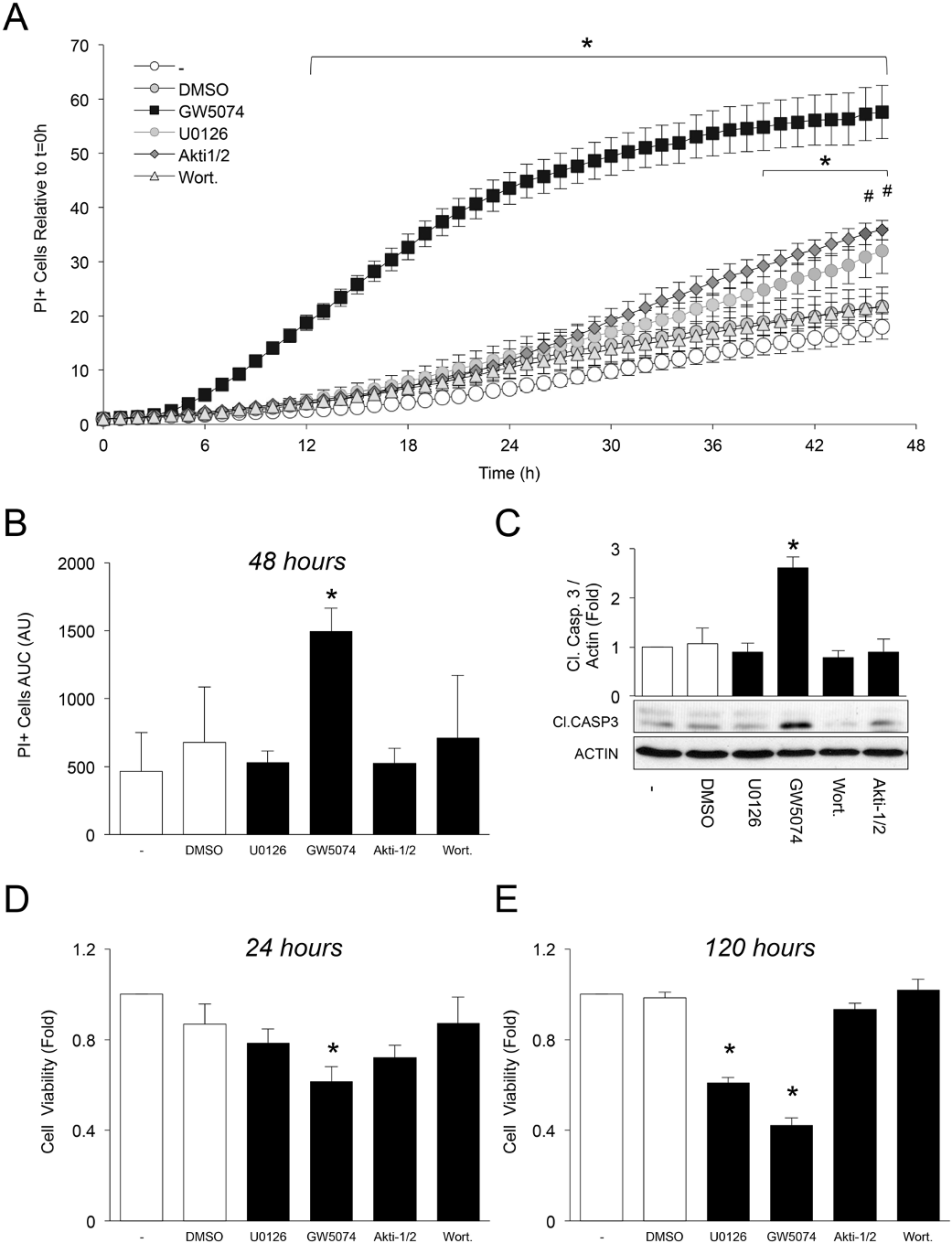
**RAF1/ERK signalling is preferentially required for PANC1 cell survival in the absence of exogenous insulin. (A)** Effects of different small molecule inhibitors on propidium iodide (PI) incorporation (PI) in PANC1 cells were tracked and expressed as the fold change in the percent of PI and Hoechst co-positive cells over total Hoechst positive cells at that hour relative to t =0 hour. Kinetic data were analyzed relative to serum-free control by two-way ANOVA (n =3) Data points that have been shaded solid black represent statistical significance when compared to non-treated conditions at that time point. # Indicates statistical significance in cells treated with Akti1/2 when compared to control at that time point. **(B)** Average number of PI positive cells over time of each treated group in Figure 5A is shown as a histogram expressed in arbitrary units (AU). GW5074 exhibited statistical significance, where as other treatments did not yield significance. U0126 *p = 0.38*, GW5074 *p = 0.0005, Akti-1/2 *p = 0.395*, Wort. *p = 0.292* (n = 3). **(C)** The effect of 24 hours treatment with inhibitors on cleaved caspase 3 protein levels in PANC1 cells. This is a representative immunoblot of three independent biological replicates (n =3). **(D-E)** PANC1 cells were serum starved and treated with either DMSO, 10 μM GW5074, 10 μM U0126, 200 nM Akti-1/2 and 1 mM wortmannin (wort.) for 24 hours and 120 hours (n =4-5). Cell viability of PANC1 cells was expressed as the fold change of the treated relative to control. (**C-E**) One-way ANOVA analysis with Bonferroni post-test was performed. *Represents statistical significance of *p* < 0.05 where treated groups are compared to control (-) in the post-hoc test.

Next, we assessed the requirement for RAF1/ERK versus PI3K/AKT signalling on the viability of PANC1 cells. Inhibition of RAF1 significantly increased cell death (Figure 5A-C) and reduced cell viability (Figure 5D,E) in PANC1 cells. A more modest delayed effect on cell viability and cell death was also observed after MEK1/2 inhibition by U0126 (Figure 5A,D,E), similar to the findings in the HPDE cells. AKT inhibition was much less effective at inducing PANC1 cell death as assessed by cell counting, PI incorporation, and cleaved caspase 3 levels (Figure 5A-E). These observations indicate that the RAF1/ERK pathway, and not the PI3K/AKT pathway, may play a more important role in the maintenance of PANC1 cell survival under these basal conditions.

### Effects of three insulin analogs on PANC1 cells

**Figure 6:**
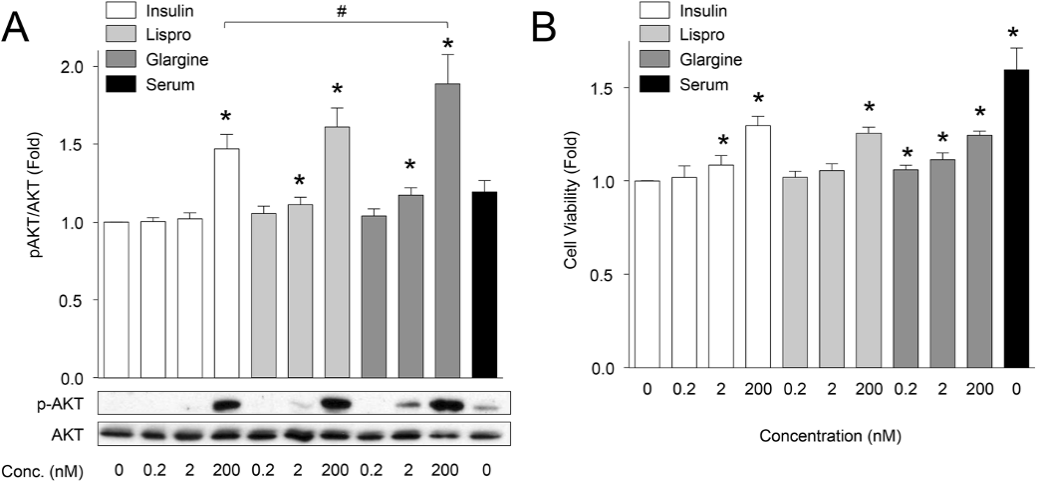
**Effects of insulin analogues on PANC1 cell viability. (A)** Effects of recombinant insulin, insulin Lispro, and insulin Glargine on AKT phosphorylation after 60 minutes (n =16). Two-tailed paired sample t-test revealed insulin Glargine promoted greater stimulation of AKT phosphorylation than recombinant insulin at the 200 nM insulin concentration denoted by # ( *p* < 0.05). **(B)** Cell viability assessed by XTT assay on PANC1 cells treated with insulin analogues for 24 hours (n =8). Two-tailed t-test were performed, and * denotes statistical significance when compared to non-treated condition.

Some studies, but not all, have reported that individuals using long-acting insulin analogs have increased risk of cancer [39]. As an adjunct to our studies on the effects of insulin in pancreatic cancer cells, we compared native insulin to a short-acting insulin analogue (Lispro™) and a long-acting insulin analogue (Glargine™) on the viability of PANC1 cells. Acute treatment of PANC1 cells with recombinant insulin, Lispro and Glargine significantly increased AKT phosphorylation (Figure 6A). No statistical difference in AKT phosphorylation was observed between the Lispro and native insulin, although our studies (n = 16) were not powered to detect very subtle differences. Glargine was found to induce slightly more AKT phosphorylation in PANC1 cells when compared to the other insulin analogues. No differences in ERK phosphorylation were observed (data not shown). Notwithstanding these modest changes in signalling, we found that recombinant insulin, Lispro and Glargine led to similar levels of PANC1 cell viability (Figure 6B). Interestingly, the viability of PANC1 cells was augmented with low doses of Glargine (Figure 6B). Together, these data indicate that all forms of insulin tested were capable of similar effects on PANC1 cell survival and proliferation, although Glargine exhibited a shift in potency. Caution should be exercised when extrapolating these *in vitro* conditions to the *in vivo* clinical situation, since high nanomolar doses of insulin are not physiologically or pharmacologically relevant.

## Discussion

Insulin and IGF1 are growth factors with putative regulatory roles in proliferation, survival and cancer progression [40]. Given that hyperinsulinemia has been identified as an independent risk factor for pancreatic cancer [1, 2, 39, 41], it is imperative to understand how changes in insulin signalling may promote cancer progression. To date, not much is known about the action of insulin on normal human pancreatic exocrine and ductal cells. Furthermore, direct comparisons of insulin signalling effects across models of different stages of pancreatic cancer have not been reported. In the present study, we demonstrated that pancreatic cancer progression is associated with changes in insulin signalling pathways that underlie cell survival, proliferation and viability. We found that primary human ductal cells are responsive to insulin and exhibit reduced cell viability when AKT signalling is disrupted. Immortalized HPDE ductal cells were also responsive to insulin, but less so than to IGF1, perhaps due to an abundance of IGF1 receptors. In contrast to the primary cells, HPDE cells required MAPK signaling and not AKT signaling to survive. The metastatic PANC1 cell model responded to insulin, more so than to IGF1, and also had a strong dependence on MAPK signalling and not AKT signalling. Collectively, our results imply a re-wiring of ductal cell dependence on the MAPK signalling axis for cell survival. Further understanding of how cells favor one pathway over another in pancreatic cancer progression may lead to novel approaches to halt early carcinogenesis and improve the long-term survival of pancreatic cancer patients.

In the present study, we found that these cell models derived from exocrine tissue required higher doses of insulin to elicit responses when compared to our previous experience with pancreatic exocrine cells that respond to physiological insulin doses in the high picomolar range [6, 26, 34, 35, 37, 38, 42]. This finding suggests the possibility that the exocrine cells and their cancerous descendants may be somewhat refractory to low concentrations insulin and may require sustained hyperinsulinemia to accelerate cancer progression. Multiple epidemiological studies have demonstrated that the hyperinsulinemic states of obesity and recent onset type 2 diabetes are associated with different types of cancer [43, 44], and this has been replicated in some animal models. For example, elevated insulin levels have been implicated in *in vivo* mouse models of breast cancer [45, 46]. The metabolic changes that result from both conditions make it difficult to discern causal factors that promote carcinogenesis. Hyperinsulinemia can precede and lead to the development of obesity [6], which suggests that it may contribute to carcinogenesis indirectly as well. Indeed, high levels of circulating insulin have been associated with increased risk of breast cancer in post-menopausal women [47, 48]. Given the association between hyperinsulinemia and pancreatic cancer [1], it has been suggested that excessive secretion of insulin by pancreatic β-cells required to maintain glucose homeostasis may directly influence pancreatic carcinogenesis in at-risk individuals.

The mitogenic actions of insulin have been well described *in vitro* and *in vivo* [49], but little is known of insulin’s proliferative effects on the endocrine and exocrine compartments of the pancreas. We previously demonstrated that insulin, even at physiological picomolar doses [7], promotes the proliferation of pancreatic endocrine β-cells [26], but whether similar effects occur on the exocrine compartment was not known. In the present study, we did not observe any proliferative effects of insulin in primary ductal cells or transformed HPDE cells. Instead, we found that insulin and closely related IGF1 promoted cell viability and survival in multiple models of pancreatic cancer progression. Collectively, these findings suggest that the oncogenic properties of insulin may be due to its effects on survival as opposed to its mitogenic effects. The downstream mechanisms of insulin action in these three models remain unclear. However, a recent report has suggested that HPDE proliferation depends on Pdx1 [50], which we have shown is an anti-apoptotic transcription factor controlled by low doses of insulin [42, 51]. Additional studies are warranted to fully elucidate the mechanisms.

## Conclusions

**Figure 7:**
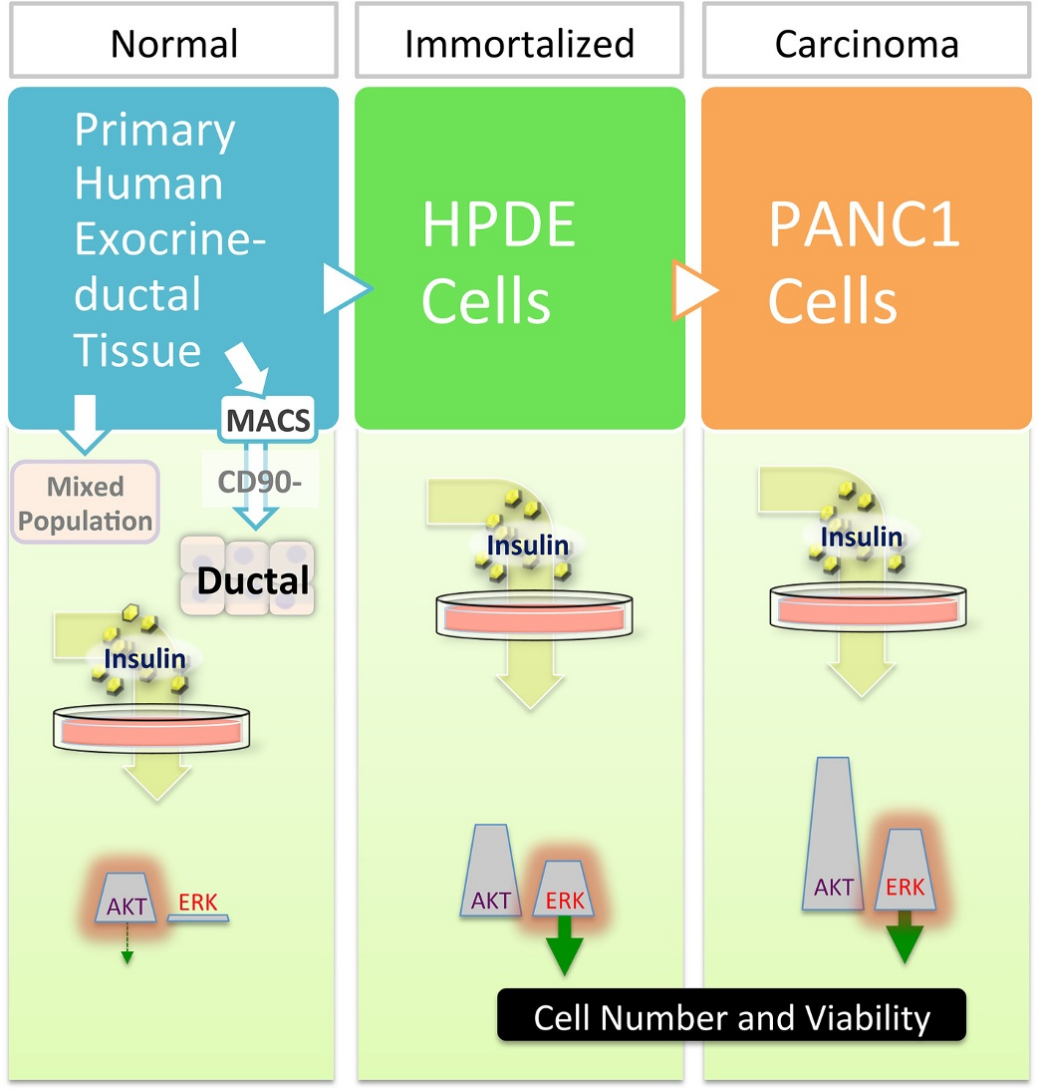
**Working model of insulin’s effects at different stages of pancreatic cancer.** Our data support a model whereby primary pancreatic duct cells respond to insulin (mostly via AKT signalling), but do not increase proliferation or survival. On the other hand, proliferative and cancerous pancreatic ductal cells respond via both AKT and ERK signalling, with cell survival predominantly controlled by the ERK pathway.

The aim of the present study was to determine whether the response to insulin was different between primary human pancreatic ductal cells, an immortalized pancreatic ductal cell line (HPDE), and an advanced pancreatic cancer cell line (PANC1). Indeed, we uncovered some interesting differences, which may hold clues to the role of insulin and insulin signalling at different cancer stages. Our data support a working model (Figure 7) whereby primary pancreatic duct cells respond to insulin (mostly via AKT signalling), but do not respond with increased proliferation or survival. On the other hand, proliferative and cancerous pancreatic ductal cells respond via both AKT and ERK signalling, with the ERK pathway being the predominant pathway controlling survival. The role of insulin during cancer progression has been debated [52–54]. The present study examined the actions of insulin on cell viability across different stages of pancreatic cancer *in vitro*. If the cell models chosen in this study faithfully recapitulate the natural progression of the disease, our experimental data may suggest that hyperinsulinemia may not play a role in initiating pancreatic cancer, but high levels of insulin may accelerate the cancer progression via increased RAF1/ERK-dependent cell survival. The studies described in this manuscript have the caveats of employing only a single cell line to represent dividing duct and adenocarcinomas and of being entirely *in vitro*. Complementary *in vivo* studies are urgently needed to assess the role of insulin and insulin signalling on pancreatic cancer progression.
